# Dietary Fructose Feeding Increases Adipose Methylglyoxal Accumulation in Rats in Association with Low Expression and Activity of Glyoxalase-2

**DOI:** 10.3390/nu5083311

**Published:** 2013-08-21

**Authors:** Christopher Masterjohn, Youngki Park, Jiyoung Lee, Sang K. Noh, Sung I. Koo, Richard S. Bruno

**Affiliations:** 1 Department of Nutritional Sciences, University of Connecticut, Storrs, CT 06269, USA; E-Mails: cmasterj@illinois.edu (C.M.); young-ki.park@uconn.edu (Y.P.); ji-young.lee@uconn.edu (J.L.); sung.koo@uconn.edu (S.I.K.); 2 Department of Comparative Biosciences, University of Illinois, Urbana, IL 61801, USA; 3 Department of Food and Nutrition, Changwon National University, Changwon 641-773, Korea; E-Mail: sknolog@changwon.ac.kr; 4 Human Nutrition Program, Department of Human Sciences, The Ohio State University, Columbus, OH 43210, USA

**Keywords:** fructose, glyoxalase I, glyoxalase II, pyruvaldehyde, rats, Sprague-Dawley

## Abstract

Methylglyoxal is a precursor to advanced glycation endproducts that may contribute to diabetes and its cardiovascular-related complications. Methylglyoxal is successively catabolized to d-lactate by glyoxalase-1 and glyoxalase-2. The objective of this study was to determine whether dietary fructose and green tea extract (GTE) differentially regulate methylglyoxal accumulation in liver and adipose, mediated by tissue-specific differences in the glyoxalase system. We fed six week old male Sprague-Dawley rats a low-fructose diet (10% w/w) or a high-fructose diet (60% w/w) containing no GTE or GTE at 0.5% or 1.0% for nine weeks. Fructose-fed rats had higher (*P* < 0.05) adipose methylglyoxal, but GTE had no effect. Plasma and hepatic methylglyoxal were unaffected by fructose and GTE. Fructose and GTE also had no effect on the expression or activity of glyoxalase-1 and glyoxalase-2 at liver or adipose. Regardless of diet, adipose glyoxalase-2 activity was 10.8-times lower (*P* < 0.05) than adipose glyoxalase-1 activity and 5.9-times lower than liver glyoxalase-2 activity. Adipose glyoxalase-2 activity was also inversely related to adipose methylglyoxal (*r* = −0.61; *P* < 0.05). These findings suggest that fructose-mediated adipose methylglyoxal accumulation is independent of GTE supplementation and that its preferential accumulation in adipose compared to liver is due to low constitutive expression of glyoxalase-2.

## 1. Introduction

**Figure 1:**
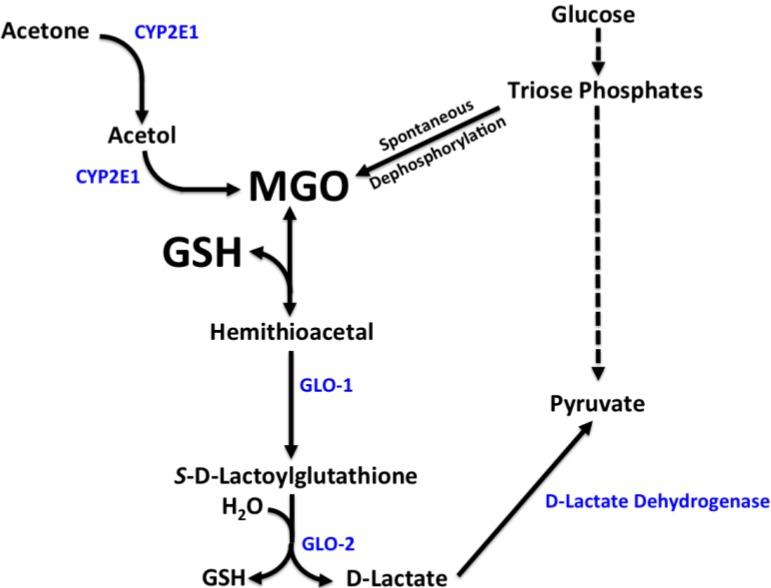
Major pathways of methylglyoxal (MGO) formation and detoxification. MGO *in vivo* is formed primarily from two pathways [2,24]. (Upper Right) triose phosphates derived from glycolysis spontaneously dephosphorylate to form MGO. (Upper Left) Acetone derived from ketogenesis is successively converted by cytochrome P450 2E1 (CYP2E1) to acetol and then to MGO. MGO detoxification (bottom left) occurs through the glutathione (GSH)-dependent glyoxalase (GLO) pathway [2]. MGO and GSH spontaneously form a hemithioacetal adduct that is successively detoxified by GLO-1 and GLO-2 to d-lactate, which is then converted to pyruvate by d-lactate dehydrogenase.

Methylglyoxal (MGO) is a highly reactive dicarbonyl and precursor to free radicals and advanced glycation endproducts (AGEs) [1]. It is formed from the spontaneous dephosphorylation of triose phosphates during glycolysis, the spontaneous fragmentation of a Schiff base during the Maillard reaction, and from ketone and threonine metabolism [2] (Figure 1). Although neither the regulation nor the pathologic consequences of MGO are fully understood, increases in MGO are associated with glucose intolerance. Indeed, individuals with type 1 and type 2 diabetes have higher plasma MGO concentrations [3]. Acute administration of MGO to rats transiently impairs glucose tolerance [4] and chronic MGO administration to rats causes β-cell dysfunction and type 2 diabetes [5], supporting a potential causal role for MGO in the development of diabetes and its complications. Moreover, overexpression of glyoxalase-1 (GLO-1) enhances MGO detoxification and protects against endothelial dysfunction in rats that is otherwise caused by streptozotocin-induced diabetes [6], suggesting that MGO detoxification reduces the risk for diabetes-related cardiovascular complications. Thus, a better understanding of the mechanisms regulating MGO accumulation will facilitate the development of novel strategies that mitigate pathogenic responses leading to diabetes and its related complications.

The extent to which MGO accumulates in tissues and plasma is dependent upon its relative rates of generation and detoxification. Once formed, MGO reacts spontaneously with glutathione (GSH) to form a hemithioacetal, which is then successively detoxified to d-lactate by GLO-1 and GLO-2 [2] (Figure 1). d-Lactate dehydrogenase then converts d-lactate to pyruvate, which can be used for gluconeogenesis, lipogenesis, or oxidation in the tricarboxylic acid (TCA) cycle [7].

The introduction of high-fructose corn syrup in 1967 led to a 30% increase in fructose consumption and a 100% increase in the consumption of free fructose [8], paralleling the rise in obesity from 13% to 34% since 1960 [9] and the subsequent rise in diagnosed type 2 diabetes from 5% to 8% since 1988 [10]. Fructose decreases insulin sensitivity in short-term clinical trials [11] and is commonly used to induce insulin resistance in animal models [12,13]. Administration of a high-fructose diet (60% w/w) to rats for nine weeks increases serum and adipose MGO while impairing adipose insulin sensitivity [12]. Co-administration of fructose and *N*-acetyl-cysteine, a precursor to GSH, abolishes these changes. Chronic fructose feeding (16 weeks) also increases serum and aorta MGO and is accompanied by lower serum GSH [14]. Although fructose would be expected to reach the highest post-absorptive concentrations in the liver, neither of these studies examined the extent to which fructose affects hepatic MGO accumulation.

No studies have directly examined the extent to which dietary fructose regulates MGO accumulation and its GLO-mediated detoxification in a tissue-specific manner. Moreover, no studies have examined whether green tea extract (GTE) prevents MGO accumulation *in vivo*. GTE is rich in polyphenolic catechins, which have been shown to trap MGO [15,16,17,18] and prevent MGO formation *in vitro* [19]. GTE also increases hepatic GSH [20], suggesting that it may enhance GSH-dependent detoxification of MGO. We hypothesized that fructose would increase MGO accumulation in liver and adipose, that differences in the degree of MGO accumulation in these tissues would correspond to differential expression of GLO enzymes, and that GTE would dose-dependently attenuate these changes. To test this hypothesis, we fed young Sprague-Dawley rats a high-fructose diet containing GTE for nine weeks. The concentrations of fructose and GTE were selected based on findings showing that fructose increases adipose MGO in Sprague-Dawley rats [12] and that GTE increases hepatic GSH [20,21]. The doses of GTE used also correspond to those associated with decreased risk of cardiovascular and liver disease in Asian populations [22]. The control diet was 10% fructose (w/w) rather than fructose-free because natural diets free of processed foods contain similar amounts of fructose from fruits and vegetables [23]. We then evaluated MGO accumulation at adipose and liver as well as the expression and activity of GLO-1 and GLO-2. Herein we report that fructose increases MGO in adipose, but not in liver, in association with low constitutive expression of GLO-2 in adipose tissue.

## 2. Experimental Section

### 2.1. Materials

HPLC grade solvents and the following chemicals were purchased from Fisher Scientific (Pittsburgh, PA, USA): diethylenetriaminepentaacetic acid (DTPA), GSH, imidazole, perchloric acid (PCA), potassium chloride (KCl), potassium hydroxide (KOH), phosphate-buffered saline (PBS), sodium acetate, and sodium phosphate buffer (NaH_2_PO_4_). The following were from Sigma-Aldrich (St. Louis, MO, USA): ethylaminediaminetetraacetic acid (EDTA), MGO, *o*-phenylenediamene (OPD), *S*-d/l-lactoylglutathione. Amyloglucosidase was purchased from Roche Applied Science (Indianapolis, IN, USA).

### 2.2. Animal Studies and Design

The protocol of this study (A08-051) was approved by the Institutional Care and Use Committee at the University of Connecticut on 2/9/11. Male Sprague-Dawley rats (5 weeks old; 240 ± 2 g; mean ± SE) were purchased from Harlan Laboratories (South Easton, MA, USA) and housed individually in a temperature- and humidity-controlled room with a 12-h light-dark cycle. Following one week acclimation, rats (*n* = 8–9/group) were assigned randomly to the following dietary groups for nine weeks as described previously [25]: a starch-based control diet containing 50% (w/w) starch and 10% fructose (w/w), a high-fructose diet containing 60% fructose, the fructose diet containing 0.5% GTE (w/w), or the fructose diet containing 1.0% GTE (Table 1). The modified AIN-93G diets, prepared as described [25], were from Dyets (Bethlehem, PA, USA) and contained egg white substituted for casein, as well as 2 mg/kg biotin, which satisfies the requirement of rats fed 20% egg white [26]. GTE was provided by Unilever BestFoods (Englewood, NJ, USA) and contained 30% total catechins (w/w), as verified by HPLC [27]. Food intake was measured daily and body mass was measured weekly.

**Table 1:** Diet Composition (g/kg) ^a^.

Ingredient	Control	Fructose	Fructose + 0.5% GTE	Fructose + 1.0% GTE
Egg whites	200	200	199	198
Corn Starch	530.7	30.7	30.5	30.4
Fructose	100	600	597	594
Cellulose	50.0	50.0	49.8	49.5
Soybean Oil	70.0	70.0	69.7	69.3
*t*-Butylhydroquinone (tBHQ)	0.014	0.014	0.014	0.014
Mineral Mix (AIN93G-EGG-MX)	35.0	35.0	34.8	34.7
Vitamin Mix (AIN-93-VX)	10.0	10.0	10.0	9.9
Biotin premix (1 mg/g sucrose)	1.8	1.8	1.8	1.8
Choline bitartrate	2.5	2.5	2.5	2.5
Green tea extract (GTE) ^b^	0.0	0.0	5.0	10.0

Diets were fed to rats (*n* =8–9/group). ^a^ Modified AIN-93G diets were from Dyets (Bethlehem, PA, USA) and contained egg white substituted for casein. ^b^ Powdered GTE was from Unilever BestFoods and was mixed into the diet as appropriate. GTE contained 30% catechins (w/w) consisting of 48% epigallocatechin gallate, 31% epigallocatechin, 13% epicatechin gallate, 8% epicatechin and was verified by HPLC-UV.

After nine weeks feeding, rats were euthanized in the fed state under isofluorane anesthesia. Blood was collected from the retro-orbital sinus into evacuated tubes containing EDTA or sodium heparin (Becton Dickinson, Franklin Lakes, NJ, USA). Plasma was obtained by centrifugation (4 °C, 15 min, 1500× *g*; Eppendorf 5810R, Hamburg, Germany), frozen in liquid nitrogen, and stored at −80 °C until analysis. Liver and epididymal adipose tissue were excised, snap frozen in liquid nitrogen, and stored at −80 °C. Portions of liver and epididymal adipose were collected into RNALater (Ambion Inc., Austin, TX, USA) for real-time PCR analysis.

### 2.3. Plasma Chemistries

Plasma glucose, cholesterol, triglycerides, β-hydroxybutyrate, alanine aminotransferase (ALT), and aspartate aminotransferase (AST) were analyzed spectrophotometrically using clinical assays (Liquid Glucose (Hexokinase) Reagent Set, Cholesterol (Liquid) Reagent Set, Triglycerides-GPO Reagent Set, β-Hydroxybutyrate Reagent Set, ALT (ASGPT) Reagent Set, and AST (ASGOT) Reagent Set; Pointe Scientific; Canton, MI, USA) on a Molecular Devices M2 microplate reader. Plasma insulin was measured using an ELISA kit (Crystal Chem Inc., Downers Grove, IL, USA) in accordance with the manufacturer’s instructions.

### 2.4. Hepatic Lipids and Glycogen

Hepatic total lipid was determined gravimetrically after overnight extraction with chloroform:methanol [27]. Following solubilization of the lipid extract, hepatic triglyceride was measured using the aforementioned assay as described [27]. Hepatic glycogen was determined by measuring glucose concentrations following glycogen hydrolysis, as described [28]. In brief, liver homogenates were prepared in ice-cold 6% PCA containing 1 mM EDTA. Glycogen was hydrolyzed by incubating 100 µL sample with 1 mL 6% PCA containing 1 mM EDTA and 20 U amyloglucosidase in 0.2 M sodium acetate (pH 4.65) for 1 h at 37 °C. The reaction was terminated by adding 0.5 mL 6% PCA containing 1 mM EDTA and samples were then centrifuged (14,000× *g*, 10 min, 4 °C). Samples were adjusted to pH 7 using a solution containing 3 M KOH, 0.3 M imidazole, and 0.4 M KCl. Following centrifugation (16,000× *g*, 10 min, 4 °C), supernatants were assayed for glucose using a hexokinase reagent kit (Pointe Scientific Inc.; Canton, MI, USA). Endogenous glucose concentrations were also measured from tissue homogenates processed in an identical manner except that the initial hydrolysis was omitted. Glycogen content was calculated by subtracting endogenous glucose levels from those obtained after hydrolysis.

### 2.5. Tissue and Plasma Methylglyoxal

MGO was measured in plasma, liver, and adipose tissue as described [29], with minor modifications. Briefly, 500 µL EDTA plasma was mixed with 100 µL PCA (0.45 M final concentration) and 20 µL OPD (10 mM final concentration) and incubated in the dark for 24 h. Following incubation, samples were centrifuged (15 min, 15,000× *g*, 4 °C) to separate precipitated proteins, and 30 µL of the supernatant were injected onto a Shimadzu Prominence HPLC system (Columbia, MD, USA) equipped with an SPD-20A detector set to 317 nm. Isocratic separation was performed at 1 mL/min for 21 min on a Nova-Pak C_18_ column (3.9 × 150 mm, 4 µm; Waters, Franklin, MA, USA) using water:acetonitrile (82.4:17.6) containing 5 mM NaH_2_PO_4_ as the mobile phase. At 21 min, the column washed by increasing acetonitrile to 50% over 1 min, holding at 50% for 1 min, and then returning to initial conditions over 1 min and equilibrating for 11 min. 2-Methylquinoxaline, the MGO derivative, was quantified by peak area relative to standards prepared in parallel. Liver and adipose tissues were homogenized 1:5 in PBS (pH 4.5) and otherwise treated as described above.

### 2.6. Hepatic and Adipose Glyoxalase-1 and Glyoxalase-2 Activities

GLO-1 and GLO-2 activities were measured as described [30], with minor modifications. In brief, liver and adipose homogenates prepared in 1:4 ice-cold 10 mM NaH_2_PO_4_ (pH 7.4) were centrifuged (30 min, 16,000× *g*, 4 °C). For GLO-1, 1.33 mM hemithioacetal was prepared by incubating 2 mM MGO and 2 mM GSH in 50 mM NaH_2_PO_4_ (pH 6.6) for 10 min at 37 °C. Liver or adipose homogenate was mixed with the hemithioacetal preparation to yield a final volume of 1 mL. GLO-1 activity was determined by measuring the formation of *S*-d-lactoylglutathione at 240 nm for 5 min at 37 °C. A value for ε_240 nm_ of 2.86 mM^−1^ cm^−1^ was used to calculate the increase in *S*-d-lactoylglutathione where 1 U of activity was defined as the amount of enzyme required to catalyze the formation of 1 µmol/min of *S*-d-lactoylglutathione. For GLO-2 activity, liver or adipose homogenate was incubated with 0.3 mM *S*-d/l-lactoylglutathione in 50 mM Tris/HCl pH 7.4 in a final volume of 1 mL. GLO-2 activity was determined by measuring the rate of hydrolysis of *S*-d/l-lactoylglutathione at 37 °C for 3 min at 240 nm. A ε_240 nm_ of 3.1 mM^−1^ cm^−1^ was used to calculate the decrease in *S*-d/l-lactoylglutathione, and 1 U of activity was defined as the amount of enzyme required to catalyze the hydrolysis of 1 µmol/min of *S*-d/l-lactoylglutathione.

### 2.7. Quantitative Real-Time PCR

Total RNA was extracted using TRIzol (Invitrogen; Carlsbad, CA, USA) according to the manufacturer’s instructions. Reverse transcription for cDNA synthesis and quantitative real-time PCR analysis were performed as described [31]. Primers were designed according to the GenBank database [32] using Primer Express software. GLO-1 was amplified using the forward primer (5′-CAGCGTGGGCTTTTTCCA-3′) and the reverse primer (5′-TCAGTGCCCCAGTTGTGTGT-3′), and GLO-2 was amplified using the forward primer (5′-AGGGAACCGCAGACGAGAT-3′) and the reverse primer (5′-GAGGAAGCCGGCCTAAGACT-3′). Data were normalized to 18S as an internal control, and subsequently normalized to the GLO-1 expression of the control group.

### 2.8. Statistical Analyses

A power analysis was performed to determine that a sample size of 8–9 was sufficient to demonstrate statistical power of 80% (*P* ≤ 0.05) to detect a 1.8 µmol/L (±0.89 SD) difference in plasma MGO [14]. All data (means ± SE) were analyzed using GraphPad Prism (version 5; San Diego, CA, USA), except that 3-way ANOVA was performed using SPSS (version 15; Chicago, IL, USA). Diet-induced changes in clinical parameters and MGO concentrations were analyzed using 1-way ANOVA with a Bonferonni correction to evaluate pairwise differences. 3-Way ANOVA was used to evaluate diet-, tissue-, and enzyme-dependent differences in the activity and expression of the GLO system, as well as all corresponding interaction effects, with a Bonferonni correction to evaluate pairwise differences. Regression analysis was performed to evaluate associations between study variables. Analyses were considered statistically significant at an α-level of *P* ≤ 0.05.

## 3. Results

### 3.1. Body Weight and Composition, Liver Injury, and Clinical Chemistries

Rats fed the high-fructose diet had less (*P* < 0.05) adipose mass compared to starch-fed controls despite no differences in food intake or body weight (Table 2). Fructose-feeding also increased liver mass, which occurred independent of any changes in hepatic lipid, triglyceride, or glycogen. Fructose increased plasma triglyceride (*P* < 0.001), consistent with the well-established hyperlipidemic effect of fructose feeding [25]. Fructose also increased plasma β-hydroxybutyrate (*P* < 0.01), but had no effect on plasma glucose, cholesterol, or insulin. GTE had no effect on any of these parameters. Plasma activities of ALT (*P* = 0.08) and AST (*P* = 0.06) tended to increase with fructose feeding and normalize to the levels of starch-fed controls when GTE was provided at either dose.

**Table 2:** Body composition and clinical chemistries ^1^.

Parameter	Control	Fructose	Fructose + 0.5% GTE	Fructose + 1.0% GTE	P
Food Intake, g/day	18.8 ± 0.5	21.8 ± 2.1	25.2 ± 1.4	26.4 ± 3.3	NS
Body Mass, g	397.0 ± 8.6	388.0 ± 29.5	404.5 ± 7.1	394.9 ± 11.3	NS
Adipose Mass, % body mass	1.48 ± 0.07 ^a^	1.20 ± 0.58 ^b,c^	1.28 ± 0.05 ^b^	1.06 ± 0.04 ^c^	0.0001
Liver Mass, % body mass	3.63 ± 0.11 ^b^	4.10 ± 0.06 ^a^	4.25 ± 0.04 ^a^	4.15 ± 0.06 ^a^	<0.001
Hepatic Total Lipid, mg/g liver	52.7 ± 3.2	52.6 ± 2.1	54.6 ± 2.4	48.7 ± 2.1	NS
Hepatic Triglyceride, µmol/g liver	26.6 ± 1.2	28.0 ± 1.5	29.1 ± 2.1	24.6 ± 1.4	NS
Hepatic Glycogen, mg/g liver	33.0 ± 1.5	34.2 ± 1.4	33.2 ± 1.4	37.0 ± 2.1	NS
Plasma Glucose, mg/dL	10.5 ± 0.9	11.6 ± 1.1	12.2 ± 0.6	13.1 ± 0.5	NS
Plasma Cholesterol, mg/dL	96.1 ± 6.0	102.6 ± 5.6	108.0 ± 4.9	104.0 ± 4.5	NS
Plasma Triglycerides, mmol/L	118.3 ± 12.1 ^b^	227.6 ± 16.5 ^a^	214.1 ± 22.6 ^a^	187.4 ± 12.3 ^a^	<0.001
Plasma β-hydroxybutyrate, mmol/L	0.265 ± 0.018 ^b^	0.423 ± 0.044 ^a^	0.441 ± 0.036 ^a^	0.353 ± 0.015 ^a^	<0.01
Plasma Insulin, pmol/L	375.5 ± 171.4	340.9 ± 153.4	260.8 ± 62.0	330.1 ± 74.5	NS
Plasma ALT, U/L	23.9 ± 4.5	41.4 ± 6.2	27.6 ± 3.8	29.5 ± 4.0	0.08
Plasma AST, U/L	46.7 ± 8.4	71.1 ± 11.5	41.1 ± 4.9	45.1 ± 5.6	0.06

^1^ Data are means ± SEM, *n* = 8–9 rats/dietary treatment. Data were analyzed by 1-way ANOVA using a Bonferonni correction to evaluate pairwise differences. Labeled a, b and c mean in a row without a common superscript differ significantly. ALT, alanine aminotransferase; AST, aspartate aminotransferases; NS, not significant, *P* > 0.05.

### 3.2. Plasma and Tissue MGO

**Figure 2:**
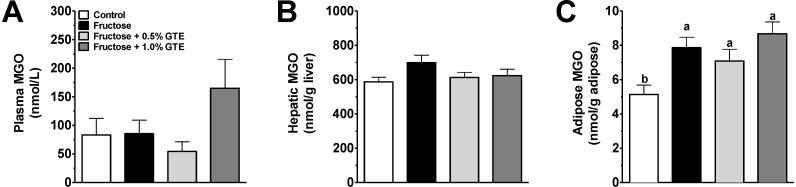
(**a**) MGO concentrations in plasma. (**b**) MGO concentrations in liver. (**c**) MGO concentrations in adipose. MGO concentrations are shown from rats fed a starch-based control diet containing 50% (w/w) starch and 10% (w/w) fructose (Control), a high-fructose diet containing 60% fructose (Fructose), the fructose diet containing 0.5% green tea extract (GTE) (w/w) (Fructose + 0.5% GTE), or the fructose diet containing 1.0% GTE (Fructose + 1.0% GTE) for nine weeks (means ± SE; *n* = 8–9/group). Samples were acidified with PCA, derivitized with OPD, and analyzed by HPLC-UV. 1-Way ANOVA main effects are significant for adipose (*P* < 0.01) but not plasma or liver (*P* > 0.05). Group means without a common superscript are different (*P* < 0.05).

Plasma and liver MGO concentrations were unaffected by fructose and GTE (Figure 2A,B). In contrast, adipose MGO concentrations increased by 53% (*P* < 0.05) in response to fructose-feeding compared to starch-fed controls, but were unaffected by GTE (Figure 2C).

### 3.3. Methylglyoxal Metabolism

**Figure 3:**
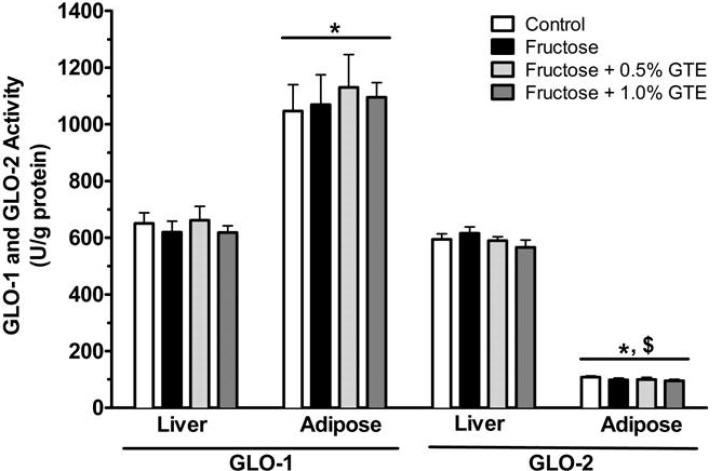
GLO-1 and GLO-2 activities in liver and adipose tissue. GLO-1 and GLO-2 activities are shown from rats fed a starch-based control diet containing 50% (w/w) starch and 10% (w/w) fructose (Control), a high-fructose diet containing 60% fructose (Fructose), the fructose diet containing 0.5% GTE (w/w) (Fructose + 0.5% GTE), or the fructose diet containing 1.0% GTE (Fructose + 1.0% GTE) for nine weeks (means ± SE; *n* = 8–9/group). Enzyme activities were measured spectrophotometrically in tissue homogenate. GLO-1 activity was determined by measuring the rate at which MGO-GSH hemithioacetal was converted to *S*-d-lactoylglutathione whereas GLO-2 activity was determined by measuring the disappearance of *S*-d/l-lactoylglutathione. Data were analyzed by 3-way ANOVA. Main effect of enzyme was significant (*P* < 0.001), but main effects of tissue and group were not (*P* > 0.05). A significant tissue by enzyme interaction was observed (*P* < 0.001), but no other significant interactions were detected (*P* > 0.05). *, significant difference between tissues for the same GLO enzyme (*P* < 0.01). $, significant difference between GLO enzymes within a tissue (*P* < 0.01).

**Figure 4:**
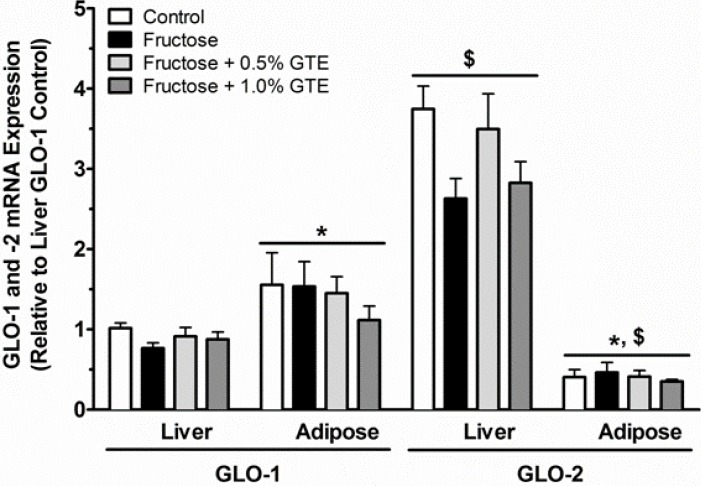
GLO-1 and GLO-2 mRNA expression in liver and adipose tissue. GLO-1 and GLO-2 mRNA expression are shown from rats fed a starch-based control diet containing 50% (w/w) starch and 10% (w/w) fructose (Control), a high-fructose diet containing 60% fructose (Fructose), the fructose diet containing 0.5% GTE (w/w) (Fructose + 0.5% GTE), or the fructose diet containing 1.0% GTE for nine weeks (Fructose + 1.0% GTE) (means ± SE; *n* = 8–9/group)***.*** RNA was isolated using TRIzol and reverse transcribed by MMLV reverse transcriptase for RT-PCR analysis using the primers described in Methods and Materials. Data were normalized to GLO-1 mRNA in the control group and analyzed by 3-way ANOVA. Main effects of tissue and enzyme as well as tissue by enzyme interaction were all significant (*P* < 0.01). There was no effect of diet (*P* > 0.05) or interaction between diet and tissue (*P* > 0.05) or group (*P* > 0.05). A significant 3-way diet by tissue by enzyme interaction was observed (*P* < 0.05), but no pairwise differences between diets were detected (*P* > 0.05). *, significant difference between tissues for the same GLO enzyme (*P* < 0.01). $, significant difference between GLO enzymes for within a tissue (*P* < 0.01).

We measured the activity and expression of GLO-1 and GLO-2 to determine whether fructose affected GLO-dependent detoxification of MGO, and whether tissue-dependent differences in MGO detoxification explained the preferential accumulation of MGO in adipose, but not in liver. Neither fructose nor GTE affected the activity (Figure 3) or mRNA expression (Figure 4) of GLO-1 or GLO-2 in either tissue. This suggests that tissue-specific MGO detoxification is not regulated in response to fructose or GTE supplementation.

Although dietary treatments had no effect on hepatic or adipose GLO-1 or -2, constitutive tissue-dependent differences in MGO detoxification could nevertheless explain the preferential accumulation of MGO in adipose. We therefore examined differences in the constitutive expression and activity of these enzymes *between tissues*. Adipose GLO-1 activity was 70% greater than liver GLO-1 activity (*P* < 0.01; Figure 3), consistent with 56% greater mRNA expression of adipose GLO-1 than liver GLO-1 (*P* < 0.01; Figure 4). In contrast, adipose GLO-2 activity was 5.9-times lower than liver GLO-2 activity (*P* < 0.01; Figure 3), consistent with 7.8-fold lower expression of adipose GLO-2 compared to liver GLO-2 (*P* < 0.01; Figure 4). These data suggest that low expression and activity of adipose GLO-2, but not GLO-1, may explain fructose-mediated increases in adipose MGO without any corresponding changes in liver MGO.

**Figure 5:**
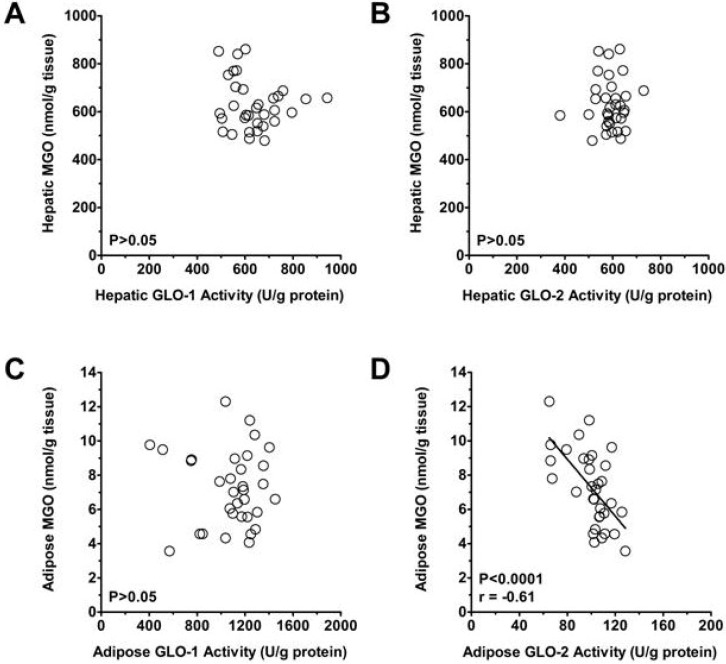
(**a**) Linear regression of MGO on hepatic GLO-1 activity. (**b**) Linear regression of hepatic MGO on hepatic GLO-2 activity. (**c**) Linear regression of adipose MGO on adipose GLO-1 activity. (**d**) Linear regression of adipose MGO on adipose GLO-2 activity. The relation between adipose GLO-2 and adipose MGO was significant (*P* < 0.0001), but the other relations were not (*P* > 0.05).

We next measured *within-tissue* differences between the expression and activity of GLO-1 and -2. At liver, there was no difference between the activities of GLO-1 and GLO-2 (Figure 3), despite 3.5-times higher GLO-2 expression at liver (*P* < 0.01; Figure 4). In contrast, GLO-2 activity was 10.8-times lower than GLO-1 activity in adipose tissue (*P* < 0.01; Figure 3), consistent with 3.4-times lower expression of GLO-2 than of GLO-1 (*P* < 0.01; Figure 4). The low expression and activity of adipose GLO-2 may explain how fructose feeding increased MGO in adipose but not in liver. In support, adipose GLO-2 activity was inversely related to adipose MGO (*r* = −0.61; *P* < 0.0001), but no relation was observed between adipose GLO-1 activity and MGO, nor was there any relation between hepatic GLO-1 or GLO-2 activity and hepatic MGO (Figure 5).

## 4. Discussion

The findings of this study show that, in support of our hypothesis, a high-fructose diet increased MGO concentrations in adipose tissue, while contrary to our hypothesis it had no effect on liver MGO and MGO accumulation at either tissue was unaffected by GTE. Regardless of dietary treatment, adipose MGO was inversely related to adipose GLO-2 activity, which was constitutively low compared to hepatic GLO-2 or adipose GLO-1. Low GLO-2 activity would be expected increase accumulation of *S*-d-lactoylglutathione, the intermediate in the GLO pathway. This, in turn, would shift the equilibrium for the reaction catalyzed by GLO-1 resulting in MGO accumulation. Our study supports earlier findings that fructose increases adipose MGO [12,14] and provides novel evidence that tissue-dependent differences in the GLO system mediate the preferential accumulation of MGO at adipose compared to liver in response to fructose feeding. We also show new evidence *in vivo* that adipose and hepatic MGO are unaffected by GTE despite existing evidence *in vitro* indicating otherwise [19]. These findings suggest that GLO-2 is an important determinant of MGO detoxification in adipose tissue and that fructose-mediated MGO accumulation is independent of supplementation of GTE.

Our finding that fructose-fed rats had lower adipose mass conflicts with the temporal association between increased consumption of fructose and the rise of the obesity epidemic [8,9], and the findings from prospective cohort studies and short-term clinical trials suggesting that sugar-sweetened beverages contribute to weight gain, overweightness, and obesity [11]. However, animal studies assessing the effect of high-fructose diets on adipose mass have been inconsistent. Some have reported that fructose increased adipose mass [33,34], while others have reported that it decreased adipose mass [25], had no effect [35], or increased adipose mass only after rats were switched from a high-fructose to a high-fat diet [36]. Our results are consistent with those of Shrestha *et al.* [25], who fed rats an identical high-fructose diet with egg whites substituted for casein and reported 60% lower adipose mass with no change in bodyweight. Why fructose-fed rats had lower adipose mass in our study is unclear. Food intake was marginally higher, but without statistical differences, among fructose-fed rats. Intestinal absorption of fructose is limited and increased by co-ingestion of glucose [37], and some [38] but not all [39] studies suggest that fructose increases energy expenditure compared to glucose. Decreased intestinal absorption or increased energy expenditure may therefore explain why fructose-fed rats had lower adipose mass. Nevertheless, fructose is not encountered as the sole carbohydrate in the diet of free-living populations and may act differently in the presence of glucose or in the context of processed foods.

Few studies have measured both GLO-1 and GLO-2 in different tissues, and we are the first to specifically define enzyme-dependent differences *within-* and *between*-tissues. Jerzykowski *et al.* [40] measured the activity of GLO-1 and GLO-2 in 18 cell lines and in the liver, kidney, brain, pancreas, heart, muscle, and spleen of 10 species, but did not measure the activity of either enzyme in adipose tissue. In rat, liver GLO-1 activity was 86% higher than liver GLO-2 activity, although no statistical analysis was reported. Kawase *et al.* [41] measured age-dependent differences in liver GLO-1 and GLO-2 in rats. Compared to GLO-2 activity, GLO-1 activity was 76% higher at five weeks, 54% higher at nine weeks, and 3.6–6.1 times higher in rats ≥2 years old, although no direct statistical comparisons were reported. Our data show that in 15 weeks old rats GLO-1 activity is only 8% greater than GLO-2 activity without any statistical significance. Larsen *et al.* [42] compared GLO-1 protein and activity in the liver and adipose of a single adult human, and found similar enzyme activities between tissues despite 3-times greater hepatic protein expression. This suggests the possibility of catalytically inactive GLO-1 in liver, which could reflect differential post-translational modifications including disulfide formation, phosphorylation, *S*-nitrosylation, and *S*-glutathionylation [43]. In contrast, the present work shows 70% higher GLO-1 activity in adipose compared to liver, consistent with 56% greater GLO-1 mRNA expression in adipose. We conducted our study in a rat model, thereby precluding a direct comparison of our results to those of Larsen *et al.* [42]. However, the differences we observed between adipose and hepatic GLO-1 activity were small. We observed much larger differences for adipose GLO-2 activity, which was 5.9-times lower than that of liver GLO-2 and 10.8-times lower than that of adipose GLO-1. These data therefore address an important knowledge gap by demonstrating the low constitutive activity of GLO-2 in adipose tissue. These findings also suggest that GLO-2 activity is a hitherto unappreciated determinant of the adipose MGO response to dietary fructose.

Although tissue-specific differences in the GLO system may explain why fructose increased MGO in adipose tissue but not in liver, they cannot explain why fructose increased adipose MGO relative to starch. Indeed, although fructose might be expected to alter GLO expression by inducing oxidative stress [44,45] and insulin resistance [45,46], the GLO system was unaffected by diet in our study. Fructose and glucose generate MGO via triose phosphates during glycolysis [47], whereas acetone derived from ketone metabolism successively generates acetol and MGO via cytochrome P450 2E1 (CYP2E1) [24]. CYP2E1 is expressed in adipose tissue [48] and undergoes protein stabilization in response to acetone [49]. Incubation of rat vascular smooth muscle cells with equimolar concentrations of fructose, glucose, or acetol results in similar increases in MGO [50]. Fructose did not increase plasma glucose in our study, but doubled plasma β-hydroxybutyrate, suggesting that an increase in ketogenesis could have contributed to adipose MGO accumulation. This is consistent with clinical studies indicating that a low-carbohydrate diet increases plasma acetone, acetol, and MGO [51], and with a study demonstrating that citrate suppresses plasma ketones and the formation of MGO-derived carboxyethyllysine in the lens tissue of diabetic rats without affecting plasma glucose [52]. Although fructose suppresses ketogenesis in humans when administered as part of a hypercaloric diet [53], weight loss increases ketogenesis [54]. Therefore, rats fed fructose in our study likely had greater ketogenesis resulting from decreased adipose mass. Nevertheless, even if acetone comprised up to 40% of plasma ketones as has been found in humans under ketogenic conditions [55,56], its concentration would remain well under that of plasma glucose and even under typical values for plasma fructose [57]. Our study was not designed to determine the source of adipose MGO, but our results support that ketone formation may have an underappreciated role in MGO generation.

Our study was also not designed to determine which GLO enzyme is rate-limiting in adipose tissue, which would have required *in situ* measurement of activity with physiologic concentrations of substrate. However, GLO-2 purified from rat has a lower kcat and higher *K*_m_ than GLO-1 [58], suggesting that GLO-2 is a less efficient enzyme and would be rate-limiting *in vivo* if the catalytically active protein content of each enzyme were equal. Our measurements of mRNA content and *in vitro* activity suggest that the amount of catalytically active GLO-2 is lower than that of GLO-1 in adipose tissue, which further supports that GLO-2 is likely rate-limiting in adipose tissue *in vivo*.

Our study may have been underpowered to detect significant decreases in plasma and hepatic lipids or significant increases in plasma MGO in rats fed 1.0% GTE, changes that would be consistent with the established hypolipidemic activities of GTE [22,25] and the recent finding that GTE increased an MGO-protein adduct in the plasma of Zucker diabetic rats [59]. The mechanism underlying the latter finding is unclear, but may be attributable to the pro-oxidant effects of GTE catechins at high concentrations or in the presence of copper ions, as well as the intracellular location of their transcriptional antioxidant effects [22], which may limit their protective activities on oxidative stress in extracellular fluids such as plasma. Nevertheless, none of the variations in these parameters reached statistical significance in our study, and may therefore be attributable to chance.

## 5. Conclusions

In conclusion, a high-fructose diet fed to male rats increases MGO accumulation in adipose but not in liver, independent of GTE supplementation, and the tissue-specific accumulation of MGO is associated with the low constitutive expression of GLO-2 in adipose. Further work is needed to characterize the mechanisms underlying tissue-specific differences in the GLO system, and to determine whether GLO-2 plays an important role in regulating tissue MGO concentrations in humans. Better defining the factors that regulate GLO-2 activity *in vivo* could lead to novel dietary or pharmacological strategies to reduce MGO accumulation and thereby mitigate the development of diabetes and its cardiovascular complications.
